# Development, Implementation, and Refinement of a Mental Health Consultation–Liaison Model in the Neonatal Intensive Care Unit: A Learning Health System Approach

**DOI:** 10.3390/bs16071249

**Published:** 2026-07-22

**Authors:** Allison G. Dempsey, Jessalyn Kelleher, Danielle L. Cooke, Susanne Klawetter, Jack Dempsey, Alejandra Santisteban

**Affiliations:** 1 Department of Psychiatry, School of Medicine, University of Colorado Anschutz Medical Campus, Aurora, CO 80045, USAalejandra.santisteban@cuanschutz.edu (A.S.); 2 UCHealth, University of Colorado Hospital, Aurora, CO 80045, USA; 3 Department of Pediatrics, School of Medicine, University of Colorado Anschutz Medical Campus, Aurora, CO 80045, USA

**Keywords:** neonatal intensive care unit (NICU), consultation–liaison, mental health, learning health system, parent–infant interactions, family engagement, mental health screening, program development

## Abstract

Proactive mental health consultation–liaison (C-L) services in neonatal intensive care units (NICUs) are increasingly recognized as essential to infant health and family functioning, yet there is limited guidance on how to develop sustainable and effective programs in real-world clinical settings. This paper describes the development, implementation, and iterative refinement of a NICU mental health C-L program using a learning health system (LHS) framework. Grounded in a theory-driven model that conceptualizes the infant as the identified patient, the program was designed to target proximal mechanisms influencing infant regulation, parent–infant interaction, and medical course. Consistent with LHS principles, program elements were introduced incrementally and refined through embedded data collection, rapid feedback loops, and multidisciplinary team learning. Data sources emphasized feasibility, reach, implementation processes, and acceptability rather than hypothesis-driven outcome testing. Over six years, this approach supported the expansion of multiple integrated service components, including dyadic intervention services, parent mental health screening and referral pathways, unit-wide family engagement initiatives, and neurobehavioral care practices. Rather than presenting a fixed protocol, this paper offers a replicable framework for developing NICU mental health C-L services that balances developmental theory, implementation realities, and sustainability. The LHS approach provides a pragmatic roadmap for NICUs seeking to build or refine mental health services in contexts characterized by clinical complexity, evolving evidence, and system-level constraints.

## 1. Introduction

Proactive mental health consultation–liaison (C-L) services in the Neonatal Intensive Care Unit (NICU) are increasingly recognized as essential for promoting the overall health of the infant and supporting the developing parent–infant relationship to optimize the long-term physical and mental health of the infant and family unit (Dineen, 2025; Kelleher et al., 2022; Kocakabak et al., 2026; Nagy et al., 2025; Osborne et al., 2025; Saxton et al., 2020; Shaw et al., 2023; Willis et al., 2022). Typically staffed by pediatric psychologists and/or psychiatrists, dedicated NICU C-L teams are uniquely positioned to intervene early by identifying infant factors that disrupt positive parent–infant interactions associated with infant health (both physical and mental) and development. Delivery of psychological interventions involving parent psychoeducation, infant self-regulation support, and/or dyadic coaching strengthens attachment, reduces stress, and promotes parental emotional regulation and infant self-regulation (Bernard et al., 2011; Mendelson et al., 2017; Shaw et al., 2023). Embedded C-L providers can deliver infant-directed health behavior interventions and other psychological services within the NICU to improve the infant’s health and care trajectory with feasible billing and documentation pathways within a pediatric setting. 

There is a growing demand for embedded C-L providers in NICU settings, and new positions are being created across the US; the demand is also emerging in additional countries, as well, such as those within the United Kingdom. However, given the relative infancy of the field (Saxton et al., 2020), creation of a new site-based program is challenging. This is particularly true in the US where psychology services are typically implemented as fee for service rather than in a universal healthcare paradigm. Frequently, medical providers from other disciplines are tasked with creating positions and establishing business proformas based on billing models to support the new service. Further, providers recruited into the new positions are expected to build effective and sustainable programs from the ground up without clear guidelines for developing NICU-specific services that are responsive to the needs of the individual unit and its patients. As an example, a review of the listserv of the National Network of NICU Psychologists indicates that in the year 2025, 11% of all new message threads were from members requesting information about how to establish specific elements of programming, including billing and documentation for NICU services, parental screening, and other logistics of setting up a new NICU service. The differing expectations and demands of the role of Pediatric C-L providers in NICUs, coupled with the rapid expansion of creation of such positions and services, has led to significant variation in practices from one NICU to the next and a lack of standardization in service delivery (both content and implementation factors). Although evidence-based interventions have emerged in the past two decades (for a review see Givrad et al., 2021), the scaling and implementation of the various components of NICU care remain challenging. Given the absence of a standardized model of NICU C-L care and the reality that the field is still “building the plane while flying it”, there is a clear need for a framework for developing or expanding programs based on fundamental principles that are applicable across NICU environments.

This paper describes a NICU-specific model of care developed in one Level III NICU based on core principles to provide effective and sustainable care to support both infants and their parents through an embedded C-L service. Core principles involved: (1) Theory-driven development with the infant (not the parents) as the identified patient, and (2) learning health systems-oriented approach with a dual focus on implementation and effectiveness. Through adoption of these principles, the program has been able to significantly grow over a six-year period and develop multiple elements of care including direct dyadic interventions, maternal mental health screening and connection to specialized outpatient care, systemic interventions to enhance family engagement, and neurobehavioral assessment and care services. 

### 1.1. Theory-Driven Development with the Infant as the Identified Patient

**Figure 1:**
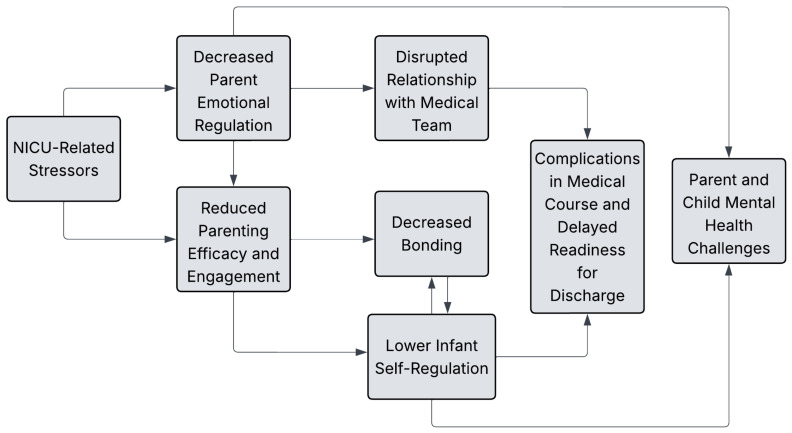
Theoretical Model of Mechanisms and Medical and Mental Health Outcomes.

Our program development and ongoing refinement and expansion are grounded in a model-based understanding of how NICU-related stress disrupts emotional regulation and relational functioning within the family system, with downstream implications for infant health (see Figure 1). This model was developed based on thorough review of the literature and constructed as an attempt to guide practice for mental health providers in NICU settings. Although the entire model has not been validated, there is significant research to support the direct associations among variables in the model. Specifically, the model describes how admission to the NICU places substantial psychological, emotional, and cognitive demands on parents at a time when they are also expected to engage in complex caregiving tasks and decision-making (Givrad et al., 2021). 

For many parents, the intensity and unpredictability of the NICU environment exceed available regulatory resources, increasing the likelihood of emotional dysregulation (Stotts et al., 2019). When parents experience emotional dysregulation, interactions with the infant and medical team can be compromised. For example, parents may avoid visitation or interactions with the medical team, rely on others to engage in infant care sessions, exhibit controlling behaviors, fixate on monitors, misinterpret infant cues, or become overwhelmed and display anger, withdrawal, or panic attacks (Gratz & Roemer, 2004; Gross, 2001). These disruptions can interfere with sensitive caregiving, limit parents’ participation in care and negatively affect the infant’s emerging capacity for self-regulation (Bridgett et al., 2015; Edwards et al., 2017; Laifer et al., 2025). Over time, maladaptive regulatory and interaction patterns may contribute to longer-term developmental and mental health vulnerabilities for the infant (Cheong et al., 2020; McGowan et al., 2022; Padrutt et al., 2025) and increase risk for perinatal mood and anxiety disorders in the parent (Lowenstein et al., 2017; Thomas et al., 2023; Verhelst et al., 2024). Parental distress and dysregulation may also affect communication and collaboration with the care team, further influencing the infant’s medical course, including feeding progression and length of stay (Melnyk et al., 2006). 

The impact on the infant’s health is central to this model because in a C-L service, the infant, not the caregiver, is the identified patient. Within this framework, the psychological services are conceptualized as intervening on proximal mechanisms (e.g., parental emotional regulation, dyadic functioning) affecting infant self-regulation and medical course rather than positioning parents as the primary patients through the delivery of individual psychotherapy for parents. This model-based understanding of risk and intervention targets informs decisions about the scope of services delivered within the NICU and provides role clarity and distinction of the service from other disciplines providing psychosocial support.

### 1.2. Learning Health System Orientation

At the outset of developing the initial C-L program, we planned to initiate services that were small and focused, with the goal that we should ultimately refine and expand programming. Thus, we laid the foundation of a learning health system (LHS) approach to establish an effective and sustainable path forward. LHS frameworks emphasize continuous cycles of data collection, reflection, and action, enabling teams to adapt services in real time and systematically develop evidence-based protocols where none yet exist in an ongoing manner (Etheredge, 2007; Friedman et al., 2015; M. Smith et al., 2012). To accomplish this, core components of LHS design include: (1) embedded, routine data capture; (2) rapid feedback loops that translate data into operational decision-making; (3) team-based learning structures; and (4) sustained iteration. Although typically implemented at a larger scale in health systems to promote quality and effectiveness, the basic LHS design components can inform the development of new programs, such as NICU C-L care models (Greene et al., 2012; Platt et al., 2020; Simon et al., 2020). LHS in mental health care emphasizes the continuous use of real-world clinical data to guide decision-making, reduce practice variability, and close the evidence-to-practice gap. It is particularly applicable in mental health systems when randomized trials are infeasible or insufficient (Stein et al., 2016). Thus, for NICU C-L models, an LHS approach can ensure: (a) that every new initiative yields learning; (b) every process is measured; (c) and every iteration brings the system and profession closer to a protocolized, sustainable, and acceptable model of care. 

Consistent with LHS principles, all new program elements begin with a data collection plan, small-scale trials of new services or workflows, and multiple cycles of iterative refinement to refine and expand the service. The clinical team meets regularly (approximately weekly) to review both informal clinical observations and formal data, using these inputs to identify barriers, assess acceptability, and guide modifications. Through successive cycles of implementation and reflection, services are refined and scaled incrementally, allowing the program to adapt to changing clinical demands and institutional constraints while maintaining alignment with the underlying developmental model. In some cases, interventions developed through this process are later evaluated using more formal research designs. In other instances, the pragmatic, data-informed refinement process is sufficient to establish sustainable services that become embedded within our routine NICU C-L team care. This approach was used within our NICU to develop and refine multiple programmatic components, including maternal mental health screening and referral pathways, unit-wide family engagement initiatives, intervention protocols and pathways, and neurobehavioral assessment and care practices.

As we develop data monitoring mechanisms and iterative refinement cycles, we seek to gather data to assess not just the effectiveness of new programing, but also the feasibility and acceptability via implementation science approaches (Trinkley et al., 2022). Central to this approach is the recognition that outcomes are not produced by the interventions alone, but by how the interventions are delivered, for whom they are delivered, and within what clinical and financial contexts. At each stage of development, we consider multiple interconnected domains (J. D. Smith et al., 2020): (a) intervention features (e.g., content, modality, and dose); (b) individual determinants of responsiveness to the intervention; (c) implementation variables such as referral pathways, timing within the hospitalization, and integration with NICU workflows; (d) proximal outcomes and hypothesized mechanisms of change; and (e) sustainability factors, including documentation practices, billing feasibility, staffing models, and institutional support. Thus, as we design and roll out new program components, we intentionally design data capture mechanisms to inform these various domains. These include the use of existing electronic health record (EHR) data, structured documentation tools such as flowsheets or spreadsheets to capture service delivery processes, and the collection of outcome data when feasible. In many cases, data collection emphasizes feasibility, reach, and process indicators rather than clinical efficacy, reflecting the program’s early developmental stage and pragmatic orientation. 

Together, these two tenants of program development provided a structured yet flexible foundation for program development. By embedding learning into routine care delivery and explicitly attending to sustainability from the outset, the framework has enabled the gradual construction of a comprehensive NICU mental health C-L program responsive to the needs of infants, families, and care teams. Thus, as previously noted, this paper seeks to describe how we applied these key principles to the development and expansion of our program as described below. Importantly, the work described here reflects quality improvement and program evaluation activities within a clinical learning health system rather than a formal research study. Consistent with this orientation, data collection was designed to inform local service development and implementation, not to generate generalizable knowledge.

## 2. Materials and Methods

### 2.1. Program Description and Setting

Funding from the state’s Health Care Policy and Finance committee was obtained in 2019 to establish inpatient programming in the hospital, as well as complementary outpatient services housed in our Department of Psychiatry’s outpatient mental health practice. This led to the establishment of the Connections Program for High-Risk Infants and Families (Connections Program) with the goal to expand access to behavioral health services for infants with medical complexities and their families during and after treatment in the NICU. Service delivery in the outpatient setting began in late 2019 and in the NICU in early 2020, right before onset of the COVID-19 pandemic. The NICU medical team deemed the services as “essential”; thus, the team was able to continue in-person service delivery without disruption, and we developed specialized programming to support families coping with the unique challenges brought by the pandemic in the unit during that time (Kelleher et al., 2022).

The C-L program was developed within a Level III NICU housed within a large academic health center hospital for adults in the state’s primary metro region. The hospital is part of the largest healthcare system in the state and is the largest Level III NICU in the metro area. The 50-bed NICU has over 650 unduplicated patients per year, approximately 350 of whom are born 34 weeks or under. It expanded its capacity in 2023, increasing to 55 beds. The payer mix is approximately 40% Medicaid and 60% private insurance. Mental health C-L services were embedded within the NICU and integrated into routine clinical care in collaboration with neonatology, nursing, social work, and allied health professionals. Pediatric psychologists and trainees with expertise in infant mental health, early childhood development, family systems, and C-L care staff the Connections Program C-L service. 

### 2.2. Team and Service Development

Implementation proceeded in phases, beginning with limited scope and gradually expanding as workflows stabilized and learning accumulated. Initially, the program started small, with a clinical director (0.2 full time equivalent (FTE)), a specialist in informatics and program evaluation (0.4 FTE), a program manager (1.0 FTE), outpatient mental health providers (psychologists, psychiatrists, social workers (3.5 FTE), and inpatient psychologists/trainees (0.6 FTE). Over time, the inpatient clinician time grew to 1.1 FTE, allowing us to consistently deliver services on the unit 5 days per week, with approximately 3.0 FTE clinician time still allotted for outpatient service delivery. Additional clinician FTE is dedicated to outpatient service delivery. To facilitate clinician engagement in program development and refinement, psychologist clinical productivity expectations are reduced by 10%. 

### 2.3. Data Sources and Data Capture Mechanisms

Consistent with LHS principles, data collection was embedded within routine clinical operations to the extent possible. At the outset of the program creation, we created a tracking system in the EHR to capture discrete data elements about specifics of each encounter. These include provider type (e.g., psychologist versus trainee), type of service (e.g., health behavior, multidisciplinary team meeting, testing), time spent in direct service delivery, time spent in indirect service delivery, and type of indirect service delivery (e.g., coordination of care, provider updates). We combined these data in a report run monthly with additional patient demographic data (e.g., sex, zip code). For each new program element, the team designed additional data capture mechanisms to track implementation variables such as feasibility, access, acceptability, and other process metrics), as well as target outcomes (e.g., satisfaction, improved parent emotional regulation). Methods of collection came from multiple formats, including the EHR, parent and staff surveys, and qualitative interviews.

### 2.4. Learning Cycles and Decision-Making Processes

In line with an LHS orientation, learning cycles were central to program development. New service elements were introduced on a small scale, followed by a period of data collection and data review. During regular meetings, the team reviewed emerging data, shared clinical observations, and identified barriers and facilitators to implementation. Decisions to modify, expand, or discontinue program elements were guided by these data-informed discussions. Over time, services that demonstrated feasibility, acceptability, and alignment with the conceptual framework and target outcomes were scaled and incorporated into routine care.

### 2.5. Ethical Considerations

Data collection activities were conducted as part of routine clinical care and ongoing program development and evaluation. These activities, including the use of clinical documentation, operational tracking systems, and family feedback (e.g., satisfaction surveys and interviews), were reviewed by the institutional review board (IRB) and determined not to meet criteria for human subjects research (#20-0442). For individual quality improvement projects, such as the development of the unit-wide family engagement initiative described below, we submitted the protocols to the IRB for additional review to ensure they did not meet criteria for human subjects research. In cases of program evaluation and quality improvement, activities were designed to support local programming rather than to generate generalizable knowledge. When data from these activities are used for research or dissemination purposes beyond program evaluation (e.g., retrospective analyses), or when prospective clinical research studies are conducted, appropriate IRB approval is obtained in accordance with institutional and regulatory requirements. This manuscript does not report research data and should be understood as a commentary describing clinical implementation and program development.

## 3. Results

### 3.1. Growth and Reach of Services over Time

The NICU mental health C-L program evolved incrementally over multiple years, with services expanding in scope, reach, and integration into routine care. Rather than launching as a comprehensive program, service components were introduced sequentially and refined and scaled over time. We broadly describe each of the core components of our service delivery below, including data collection elements and major iterations. Consistent with the program’s developmental stage and learning health system orientation, data reported here focus primarily on service growth, reach, and implementation processes rather than formal quantitative evaluation of clinical outcomes. Table 1 summarizes selected learning cycles across program development, illustrating how identified needs, program adaptations, and data sources informed successive refinements. Notably, the description of the unique data for each initiative and how the data drove iterations is beyond the scope of this paper, but many have been published in more detailed descriptions of our specific program elements (e.g., Kelleher et al., 2026).

**Figure 2:**
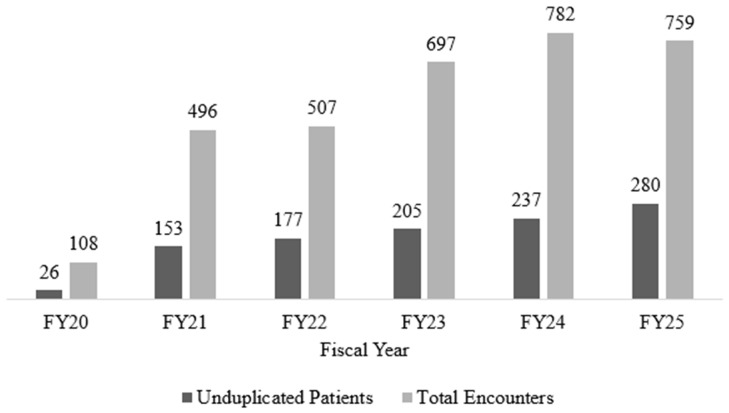
Connections Program Reach by Fiscal Year, FY20–FY25.

**Figure 3:**
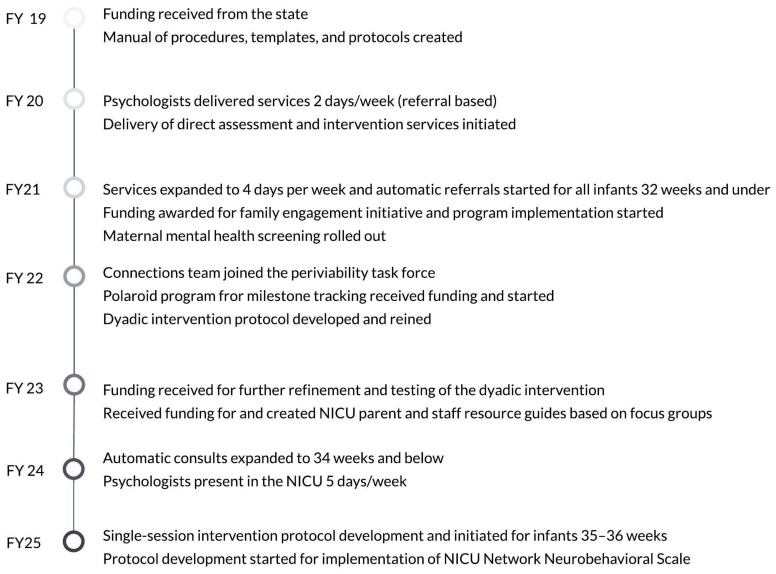
Timeline of Program Development Across Fiscal Years, FY20–FY25.

Across the program’s development, there was steady growth in the number of infants and families who received services from the C-L team (see Figure 2). Importantly, these numbers reflect individual service delivery and do not capture reach based on unit-wide initiatives or other activities (e.g., maternal mental health screening, participation in multidisciplinary rounds, systems level interventions). There are small variations within and across years due to staffing changes associated with prolonged leave, gaps in staffing, and other routine challenges to staffing clinical services. We developed productivity expectations for our NICU clinicians and reviewed encounters weekly to problem-solve challenges or facilitators to meeting expectations and to make sure current staffing models were appropriate. Broadly, for each half-day dedicated to NICU clinical time, the C-L providers are expected to spend approximately two hours in face-to-face direct service delivery, one hour in indirect service activities at the patient level, and one hour in unit-based or other administrative activities. Figure 3 depicts a summary of the evolution of the program across the years of its development. 

### 3.2. Intervention Services

As the program matured, referral sources diversified, reflecting increased awareness and acceptance of the C-L role among NICU staff. We initiated direct service delivery in February 2020. At first, referrals were primarily initiated by a limited number of disciplines or in response to acute psychosocial concerns. Over time, referrals increasingly originated from nursing, neonatology, and allied health providers and were initiated earlier in the hospitalization. We developed a process for automatic consults to be placed to our team for all babies born 30 weeks gestation on earlier in fall of 2020 and expanded this to 32 weeks and below in early 2021. 

In 2021 we began rollout of a protocolized dyadic intervention and simultaneously collaborated with hospital and unit leadership to create workflows for offering services to parents via telehealth from the infant’s bedside. Our team also joined our unit’s periviability taskforce in early 2022, to develop care pathways and process for infants delivered at 22–23 weeks gestational age starting prior to delivery. These infants and families were then able to be supported by our team shortly after the initial neonatology consult through inpatient admission, as well as in our outpatient psychotherapy program after discharge as needed. We further expanded routine services to infants born 34 weeks and below (2024) and then to 36 weeks and below (2025) with the launch of a single-session dyadic protocol for late preterm infants. When the state Medicaid program began reimbursing health and behavior codes in 2025, we worked closely with our revenue cycle management and coding teams to refine documentation for optimized reimbursement.

### 3.3. Parent Mental Health Screening and Referrals

In parallel, the range of service activities expanded to include universal maternal mental health screening and referral pathways, first implemented in June 2020 with an initial cadence of 2 weeks, 1 month, 2 months, 4 months, and 6 months postpartum. This cadence was refined over time, including elimination of the 2-week time point and the introduction of telehealth screening workflows in 2021 to improve reach and feasibility.

Screening measures are administered and scored by a mental health clinician, typically in person in the NICU, allowing for immediate triage. Resources and referrals are offered to 100% of patients with a positive screening score. Clinicians document screening scores in the mother’s medical record and, when applicable, indicate triage status and plan. To protect parent privacy, details of screening results and triage are not included in the infant’s chart; documentation is limited to whether screening was completed.

We engaged in continuous iterative refinement to improve adherence to screening protocols and reduce missed screenings, particularly for parents who were not consistently available at the bedside or faced barriers to standard workflows such as limited literacy, language differences, or cognitive challenges. These refinement cycles led to development of telehealth screening and multilingual workflows in 2021, improved tracking and communication around upcoming and completed screening time points in 2022, adoption of alternative verbal screening approaches for parents with limited literacy or cognitive ability in 2025, and expansion of screening to include anxiety in addition to depression in 2025.

### 3.4. Unit-Wide Family Engagement

As the Connections Program grew in scope and integration, NICU medical and nursing leadership identified family engagement as a priority area where the program could extend its impact. In response, we partnered with the NICU medical director and nurse manager to support unit-wide efforts to strengthen parent involvement in care through a series of family engagement initiatives.

These initiatives were implemented in a staged manner and included a family engagement telehealth intervention (launched in 2021; Kelleher et al., 2026), an “All About Me” program to encourage parents to reflect on their infant’s emerging personality and create a bedside poster (2022), a Polaroid program to help families capture milestones during the NICU stay (2022), development of guides for parents and staff outlining activities to promote infant parent interaction (2023), and introduction of developmentally appropriate materials such as rattles and black and white books to support engagement as infants approached discharge (2024).

### 3.5. Neurobehavioral Services

In the NICU environment, rounds are a critical component of multidisciplinary care and collaboration. Our team established weekly “Developmental Support Rounds” focusing on addressing barriers to family engagement and developmentally appropriate care (2022). Through participation in rounds, the rounding team (including developmental therapists and the C-L team) identified a need for more thorough assessment of infants’ state regulation, stimulation needs, and soothability, along with enhanced parent coaching and guidance to support parents’ responses to these needs, particularly as infants approached discharge. Thus, in early 2025 the team began developing workflows for routine standardized assessment of neurobehavior and development with the NICU Network Neurobehavioral Scale 2nd Edition (Lester et al., 2026) and the Bayley Scales of Infant Development—4th Edition (Bayley & Aylward, 2019). These protocols include associated coaching to parents based on assessment results.

## 4. Discussion

NICU mental health services are increasingly recognized as essential, yet there remains substantial variability in how such services are conceptualized, implemented, and sustained (Dempsey et al., 2022; Osborne et al., 2025; Pineda et al., 2023). Many existing models emphasize service delivery focused on parent mental health, often without sufficient attention to system constraints or implementation feasibility. While the detection of parent mental health concerns through screening and referral for mental health is an essential component of NICU mental health care (Hall et al., 2015; Hynan et al., 2015; Johnson Rolfes & Paulsen, 2022), the delivery of mental health services to address parental mental health (i.e., psychotherapy) in a NICU is complicated by multiple implementation factors. There are significant logistical challenges to providing mental health services to non-patients, including legitimate concerns regarding informed consent, documentation, confidentiality, and billing (Ahmed et al., 2026; Morris et al., 2026). Instead, by targeting proximal mechanisms (e.g., parental regulation and parent–infant interaction) to improve infant health within the NICU context, the C-L model reframes mental health care as an integral component of infant-centered medical care rather than an ancillary service for parents (Givrad et al., 2021; Lakatos et al., 2024; Shaw et al., 2023).

The evolution of this novel NICU mental health C-L program underscores why an LHS approach is particularly well-suited to NICU C-L program development. Traditional models of intervention development that rely on fixed protocols and delayed outcome evaluation, may be insufficient or impractical for building new programs. Thus, rather than evaluating outcomes from a single intervention or protocol, we focus on the process by which a sustainable, integrated model of care was incrementally built. In a recent review of areas for growth for C-L programs and providers, Yost and Cavanagh (2025) highlight the need for C-L providers to be able to adapt services so that they can be responsive to needs for innovation and ways to be more effective in the ever-changing healthcare landscape. Our findings highlight the value of an infant mental health model-based program guided by an embedded data infrastructure focusing on both implementation and effectiveness that allows for flexibility. Our adoption of the LHS approach with phased implementation and ongoing refinement allowed our team to build multiple program elements responsive to the specific needs of our unit. The use of routinely collected data and qualitative feedback supported timely decision-making and reduced reliance on resource-intensive research designs and helped us build services that were not only clinically relevant, but also sustainable (Trinkley et al., 2022). In doing so, it balanced the need for rigor with the realities of clinical care delivery, offering a model for us to build innovative programming in a setting where randomized trials would not be feasible (Friedman et al., 2015). Other C-L programs have demonstrated how strong data collection across a program’s lifespan can provide key data to describe practice patterns and communicate value (Piazza-Waggoner et al., 2013).

A key contribution of this work is the explicit integration of sustainability considerations into program design from the start. By attending early to billing and documentation, EHR integration, and workflow development, the Connections program was able to scale gradually while maintaining operational viability. This systems-oriented approach also facilitated our broader integration and collaboration with other NICU care teams. Overtime, we were therefore able to broaden services and increase the reach of the program. Such collaboration is critical for scalability across institutions, where buy-in from interdisciplinary teams and alignment with existing structures are often critical components for sustainability and effectiveness (Cuttini et al., 2020).

The framework presented in this paper offers a practical roadmap for providers seeking to develop or refine mental health C-L services in their NICUs. Key implications include the value of beginning with a clear model with the infant as the identified patient building and refining program components in iterative cycles, and prioritizing data infrastructure that supports improved implementation rather than solely outcome evaluation. Programs may benefit from resisting the pressure to launch comprehensive services prematurely and instead adopt a phased approach that allows for adaptation and growth to meet the needs of their unit. In addition, this approach facilitates role clarity about the type of services that are delivered by C-L providers, which is a critical element to consider in building a new program (Saxton et al., 2020; Willis et al., 2022). C-L providers are uniquely positioned to support infant development and family functioning through infant- and dyad-directed interventions that align with medical care goals. Clear articulation of this role may help address common misconceptions about NICU mental health services and support advocacy for appropriate staffing and resources.

### 4.1. Implications for Research and Policy

From a research perspective, this paper contributes to a growing recognition that program development and implementation processes are themselves worthy of systematic study (Trinkley et al., 2022). Few NICU-based C-L programs have been described in the literature and those that do exist focus on description of the services rather than how they evolved over time (Lakatos et al., 2024). By documenting how services evolve over time pragmatically, this work complements traditional efficacy-focused research and provides essential structure and context for interpreting future outcome studies. The framework described here may also inform the design of multi-site collaborations and comparative effectiveness studies by offering a shared developmental and implementation language. 

At a policy level, the findings underscore the need for reimbursement structures and quality metrics that reflect the realities of NICU mental health care. Current models often prioritize individual therapy or screening outcomes related to parental mental health, potentially undervaluing consultation, dyadic intervention, and systems-level contributions of NICU C-L teams (Ahmed et al., 2026; Morris et al., 2026). Aligning policy incentives with model-informed, sustainable models of care is needed for expanding access to effective NICU mental health services nationally. Application of this framework in other NICU settings will require adaptation to local clinical, operational, and financial contexts.

### 4.2. Limitations and Future Directions

This paper reflects the experience of one C-L program implemented in a single Level III NICU and does not capture all contextual factors influencing program development in other NICU settings. As such, the manuscript does not include hypothesis-driven analyses, comparator conditions, or formal evaluation of clinical effectiveness, which limits the ability to attribute observed changes to specific interventions. Rather, the focus is on describing the processes and iterative development that supported implementation within a real-world clinical setting.

Because this paper describes data collected as part of routine development and evaluation of services and quality improvement, it is not generalizable and does not meet the criteria for human subjects research per Department of Health and Human Service regulations. Thus, the findings are not meant to be replicable. In addition, variability in NICU structure, staffing models, reimbursement environments, and institutional priorities may influence the feasibility and adaptation of this model in other settings. As such, the framework is not intended to be directly generalizable, but rather to provide a set of guiding principles that can be adapted to local context.

Additionally, while descriptive and process-oriented data provide valuable insights for program development and refinement, they do not substitute for formal evaluation of clinical outcomes. We did not conduct hypothesis-driven analyses, comparator-based evaluations, or statistical assessments of outcomes, and therefore cannot draw conclusions regarding clinical effectiveness. This reflects the program’s emphasis on iterative development and implementation within routine care. Accordingly, the focus of this manuscript is on describing the processes and system-level refinement that supported implementation in a real-world clinical setting.

Future work should examine how LHS-developed NICU mental health programs influence infant, parent, and system-level outcomes across diverse institutions. Nonetheless, the framework described here provides a foundation for such work by articulating a replicable approach to program development that can be adapted to individual NICUs. Notably, the focus of this paper was on the development of a program within the US and with its unique socio-economic and healthcare factors shaping it. Although NICU psychologists in other countries may face differing factors that shape the development of their care, the application of a learning health system approach may still be applicable to shaping their services.

### 4.3. Conclusions

This paper demonstrates how an LHS approach can support the stepwise development of sustainable, model-informed NICU mental health C-L programs. By grounding services in a clear conceptual model, embedding learning into routine care, and balancing collection and evaluation of implementation and outcome data, this approach offers a pragmatic path forward for advancing NICU mental health care.

## Figures and Tables

**Table 1:** Program Components, Data Elements, and Major Iterations.

Program Component	Data Elements (Process/Implementation)	Data Elements (Outcomes)	Major Iterations
Direct Assessment and Intervention Services	Acceptability Parent satisfaction Feasibility of delivery	Parenting efficacy Parent sensitivity to infant cues Parent emotional regulation Infant self-regulations	Automatic consults Protocolized dyadic intervention development Incorporation of telehealth workflows Periviability workflows Documentation and billing optimization Late preterm single-session protocol development
Parent Mental Health Screening	Proportion screened Screening Language Use of interpreter Reason for not screening	Scores Triage Status Referrals	Cadence and exclusions (if currently in therapy) Workflows for non-English speaking parents Reminders for when screening is due Inclusion of anxiety screening
Unit-Wide Parent Engagement	Logs of staff tablet use Reports regarding technical quality of calls Parent satisfaction Provider satisfaction	Parent engagement in care sessions Unit-wide family engagement survey results	Development of workflows with tablets and telehealth platform All About Me staff and parent guides for encouraging bonding Polaroid program for milestone tracking
Neurobehavioral Care	Regularity of provider group attendance in rounds Parent satisfaction	Neurobehavioral scores Infants receiving developmental follow-up after discharge	Developmental rounds structure and format Referral workflows for outpatient follow-up Neurobehavioral testing protocol and workflows

## Data Availability

Data described in this manuscript were derived from existing clinical and operational systems and are not publicly available due to privacy and institutional restrictions.
